# Hybrid Nanostructures Containing Sulfadiazine Modified Chitosan as Antimicrobial Drug Carriers

**DOI:** 10.3390/nano6110207

**Published:** 2016-11-10

**Authors:** Bogdanel Silvestru Munteanu, Raluca Petronela Dumitriu, Lenuta Profire, Liviu Sacarescu, Gabriela Elena Hitruc, Elena Stoleru, Marius Dobromir, Ana Lavinia Matricala, Cornelia Vasile

**Affiliations:** 1 Department of Physics, Faculty of Physics, “Al. I. Cuza” University of Iasi, 11 Carol I bvd, 700506 Iasi, Romania; muntb@uaic.ro (B.S.M.); marius.dobromir@uaic.ro (M.D.); 2 Department of Physical Chemistry of Polymers, “P. Poni” Institute of Macromolecular Chemistry, Romanian Academy, 41A Grigore GhicaVoda Alley, 700487 Iasi, Romania; rdumi@icmpp.ro (R.P.D.); elena.paslaru@icmpp.ro (E.S.); 3 Department of Pharmaceutical Chemistry, Faculty of Pharmacy, “Grigore T. Popa” University of Medicine and Pharmacy, 16 University Street, 700115 Iasi, Romania; 4 Department of Inorganic Polymers, “P. Poni” Institute of Macromolecular Chemistry, Romanian Academy, 41A Grigore GhicaVoda Alley, 700487 Iasi, Romania; livius@icmpp.ro; 5 Department of Polymer Materials Physics, “P. Poni” Institute of Macromolecular Chemistry, Romanian Academy, 41A Grigore GhicaVoda Alley, 700487 Iasi, Romania; gehitruc@icmpp.ro (G.E.H.); vasiliu.lavinia@icmpp.ro (A.L.M.)

**Keywords:** chitosan, sulfadiazine, electrospinning, nanostructures, release kinetics

## Abstract

Chitosan (CH) nanofibrous structures containing sulfadiazine (SDZ) or sulfadiazine modified chitosan (SCH) in the form of functional nanoparticles attached to nanofibers (hybrid nanostructures) were obtained by mono-axial and coaxial electrospinning. The mono-axial design consisted of a SDZ/CH mixture solution fed through a single nozzle while the coaxial design consisted of SCH and CH solutions separately supplied to the inner and outer nozzle (or in reverse order). The CH ability to form nanofibers assured the formation of a nanofiber mesh, while SDZ and SCH, both in form of suspensions in the electrospun solution, assured the formation of active nanoparticles which remained attached to the CH nanofiber mesh after the electrospinning process. The obtained nanostructures were morphologically characterized by scanning electron microscopy (SEM) and atomic force microscopy (AFM). The SDZ release profiles and kinetics were analyzed. The SDZ or SCH nanoparticles loosely attached at the surface of the nanofibers, provide a burst release in the first 20 min, which is important to stop the possible initial infection in a wound, while the SDZ and SCH from the nanoparticles which are better confined (or even encapsulated) into the CH nanofibers would be slowly released with the erosion/disruption of the CH nanofiber mesh.

## 1. Introduction

Due to the nanoscale dimensions, high porosity, high surface/volume ratio and hence high surface exposed to the release media, electrospun/electrosprayed nanoporous structures provide very short diffusion length [1,2] and more efficient mass transfer [3] for drug release in comparison with drug-loaded films. In addition, electrospun/electrosprayed nanofibers/nanoparticles (NF/NP) can be coated onto various substrates which have the required flexibility to conform to irregular wound surfaces.

According to literature data, the active substances (AS) can be either enclosed inside the individual polymeric NF/NP or entrapped (usually in form of NP) in the NF mesh. The enclosing is performed either by coaxially electrospinning of the AS and the polymer [4] or by mono-axially electrospinning the polymer/AS blend solution. The entrapment of AS as functional NP, in the NF mesh can be realized by spraying the NP suspension onto the NF mesh [5], or electrospraying the NP onto previously electrospun nanofibers [6]. The attachment/sticking of the entrapped functional NP to the electrospun NF scaffold [7] can be realized by spraying the NP suspension into the electrospinning jet so that the particles are attached to the fiber surface prior to deposition on the mat [8].

Simultaneous electrospinning and electrospraying [9], coaxial electrospinning (active substance and polymer separately fed through two concentric nozzles) [10] and even emulsion (mono-axial) electrospinning (polymer/active substance blend solution fed through a single nozzle) [11] can also be used to obtain NF/NP structures with attached functional NP onto the surface of the NF (hybrid NF/NP nanostructures). In this respect, cells were incorporated into fiber scaffolds by simultaneous electrospinning of the fibers and bio-electrospraying the suspension of cells [12]. Also, coaxial electrospinning with the electrospinnable (NF forming) polymer solution flowing through the central nozzle and of the colloidal suspension of the NP through the outer nozzle generates NF covered with NP, co-deposited from colloidal suspension during the process of electrospinning [10].

An advantage of the NF/NP structures with attached functional NP onto the surface of the NF is the improved availability of the active substances to the targeted medium, as was found for the biocomposite nanofibrous scaffolds obtained by the electrospraying of hydroxyapatite NPs onto previously electrospun poly(l-lactic acid)-*co*-poly(3-caprolactone) nanofibers which proved to be more suitable for bone tissue regeneration in comparison with hydroxyapatite blended nanofibers [13].

Besides the aforementioned increased availability, the obtaining of such structures by electrospinning may help sticking of the active substances onto the electrospun nanofibers. Hybrid polylactic acid (PLA) nanofibrous structures containing ZnO microparticles stuck onto the PLA fiber surface were obtained by simultaneously: electrospinning a fiber forming PLA solution and electrospraying a ZnO suspension in which a small amount of PLA (well below the concentration needed for electrospinning) was dissolved to help sticking of the electrosprayed ZnO particles onto the electrospun PLA fibers [14]. Due to the availability of the ZnO microaggregates electrosprayed on the PLA fibers surface, the hybrid fibers had higher antibacterial activity than the corresponding PLA fibers contained inside ZnO particles. Post-spinning electrospraying forms a denser deposit of particles on the surface of the mat, while in simultaneous electrospinning-electrospraying the particles are more uniformly distributed within the fiber layers [15].

In wound healing applications electrospun NF mesh can be chosen to help skin reconstruction, while the drug within the NP entrapped into the NF mesh will be released when the NF mesh is swollen, biodegraded [6], and/or absorbed by the human body. A successful wound dressing system will give a large initial burst release which is important to deal with the immediately invading bacteria, followed by a sustained release at an effective inhibitory level [16]. The burst release can be easily achieved when a major part of the NP entrapped into the NF mesh is exposed at the fiber surface [17].

Chitosan (CH) nanofiber mesh is a good candidate for wound healing systems due to chitosan biocompatibility, antibacterial, and antifungic [18] activities. In addition, chitosan enhances the wound healing [19] favoring fibroblast attachment [20] and re-epithelialization [21] of the wound.

There are studies about combining chitosan with sulfadiazine (SDZ) which is a well-known antibacterial agent [22] used in the treatment of wound infections [23]. In this regard, electrospun chitosan/polyethylene oxide [24] and chitosan/polyurethane [25] nanofibers containing silver sulfadiazine had antibacterial activity against both Gram-negative and Gram-positive bacteria. The silver sulfadiazine release from various chitosan matrices was also studied. In acidic medium the release from chitosan beads is governed by chitosan erosion [26] or even disintegration [27]. The release from chitosan/chondroitin sulfate films at neutral pH had a sustained release (days) [28].

In our previous paper, the higher antibacterial activity of porous membranes obtained by lyophilization of sulfadiazine chitosan derivative (SCH) [29] and of polyelectrolyte complexes containing SCH [30] was demonstrated compared with unmodified chitosan. Furthermore, cell proliferation and MTT (3-(4,5-dimethylthiazol-2-yl)-2,5-diphenyltetrazolium bromide) tests proved their good biocompatibility and the in vivo study using a burn wound model induced in Wistar rats demonstrated an improved healing effect and enhanced epithelialization of the wound in the presence of the chitosan-sulfadiazine derivatives compared to neat chitosan [31].

In this work, hybrid NF/NP stuctures containing high molecular weight CH combined with SDZ or SCH were obtained both by mono-axially electrospinning SDZ/CH blend solution or coaxially electrospinning of CH and SCH solutions. To our knowledge there are no studies concerning the sulfadiazine release from electrospun/electrosprayed hybrid nanostructures of chitosan or chitosan derivatives.

It is known that only CH with sufficient high molecular weight can produce bead-free nanofibers when dissolved in concentrated acetic acid solution [32]. Thus, the fiber forming high molecular weight CH assures the formation of the NF mesh while the SDZ or SCH both in the form of a relatively stable suspension assure the formation of the active NP attached to or encapsulated into the CH nanofiber mesh. The obtained nanostructures were morphologically characterized by SEM (Scanning Electron Microscopy), TEM (Transmission Electron Microscopy), and AFM (Atomic Force Microscopy). The sulfadiazine release profiles and kinetics were analyzed.

## 2. Experimental

### 2.1. Materials

Sulfadiazine (4-amino-*N*-(pyrimidin-2-yl)-benzene-sulfonamide) (SDZ), chitosan (CH) (from crab shells with an average molecular weight of 310,000–375,000 g/mol) were obtained from Sigma-Aldrich, Schnelldorf, Germany. Glacial acetic acid (analytical purity) was obtained from Chemical Company, Iasi, Romania. Sulfadiazine-chitosan derivative (SCH) synthesized according to the method described in Reference [30] has a substitution degree of 31% (determined by ^1^H NMR as the ratio between the integral of the peak of the four aromatic protons from sulfonamide and integral of the characteristic peak of proton from –CH–NH_2_ group of chitosan [31]). It was demonstrated that between chitosan and sulfadiazine both ionic and covalent bonds can be formed [30].

### 2.2. Preparation of the Hybrid Nanostructures

**Scheme 1:**
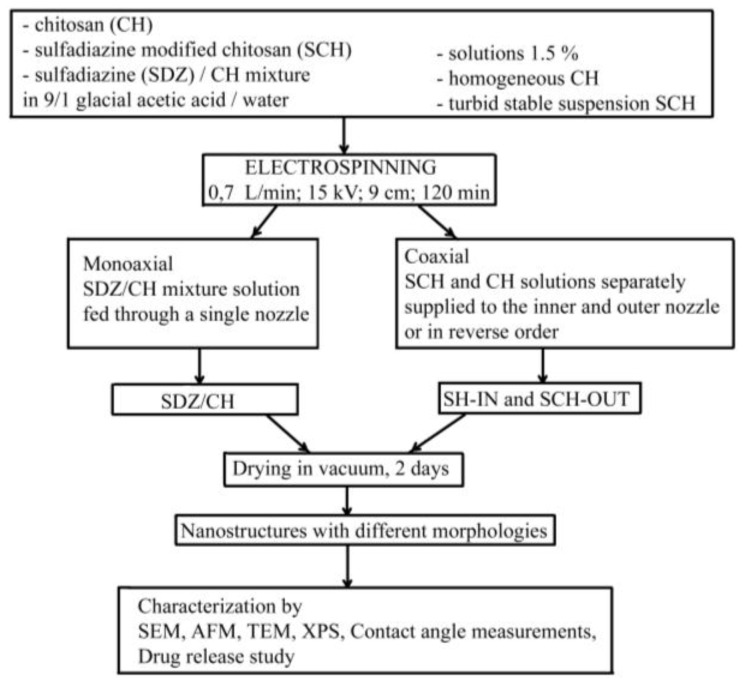
Experimental set-up for the obtaining of different nanostructures based on chitosan (CH) and modified chitosan (SCH).

The experimental set-up is presented in Scheme 1.

**Figure 1:**
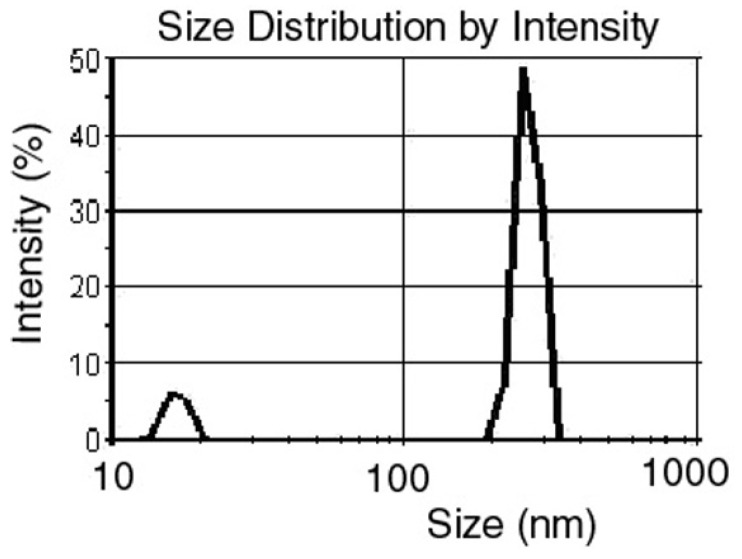
Particle size distribution of the sulfadiazine-chitosan (SDZ/CH) derivative suspension.

The CH, SCH and SDZ/CH solutions used for electrospinning were prepared in 9/1 glacial acetic acid/water solvent with 1.5 wt % polymer concentration. The SDZ/CH mixture had the composition 31/69 wt % SDZ/CH to match the SDZ content in the SCH derivative. The CH easily solved in the acetic acid giving a high viscous solution with a dynamic viscosity higher than 400 mPa·s at 20 °C, while SCH had low solubility in the acetic acid solution, forming a turbid stable suspension which maintained its aspect even after two days or more. The stability of the SCH suspension was sustained by dynamic light scattering (DLS) analysis which showed a main stable particle population (~250 nm Z-average size and ~20 nm half-width) (Figure 1). Its zeta potential (+20 mV) was within the stability limit for suspensions. The SDZ component in the SDZ/CH mixture had also low solubility, but the high viscosity of the CH component solution inhibited SDZ sedimentation.

Electrospinning was used to produce chitosan nanostructured/nanoporous morphologies containing SDZ or SCH. In order to enclose/entrap SDZ or SCH as nanoparticles into the CH nanofiber meshes, one mono-axial and respectively two coaxial electrospinning designs were used. It is well-known that the most part of the solvent evaporates during the electrospinning process [33]. However, to be sure of complete solvent removal, the electrospun nanostructures were placed for two days in a vacuum desiccator (Binder GmbH, Tuttlingen, Germany). Complete removal of the acetic acid was checked by attenuated total reflectance infrared (ATR-FTIR) spectroscopy (using a Bruker VERTEX 70 spectrometer, Ettlingen, Germany). In the spectra of the nanostructures many bands are overlapped, however the three sharp characteristic bands of acetic acid in the fingerprint region namely: C=O at 1721 cm^−1^, –OH at 1419 cm^−1^ and –C–O at 1296 cm^−1^ (NIST WebBook—National Institute of Standards and Technology) do not appear anymore. The mono-axial design consisted of the SDZ/CH mixture solution being fed through a single nozzle (sample designated as SDZ/CH). The coaxial design consisted of SCH and CH solutions separately supplied to the inner and outer nozzle (and vice versa) (samples designated as SCH-IN and SCH-OUT respectively). The optima electrospinning parameters used for all samples are presented in Table 1.

Due to the very low solubility of SDZ in the acid acetic solution it was not possible to obtain coaxial electrospun samples with SDZ solution fed through the inner nozzle and the CH solution fed through the outer nozzle. In order to obtain a 31/69 wt % SDZ/CH composition in the final dry nanostructures it would be necessary to have a SDZ concentration much over the SDZ solubility limit which would lead to SDZ sedimentation in the inner syringe during the electrospinning process (which takes tens of minutes). On the contrary, the SDZ sedimentation did not take place for the SDZ/CH solution mixture due to the high viscosity of the chitosan solution.

### 2.3. Characterization of the Solutions and Hybrid Nanostructures

Dynamic Light Scattering (DLS) of the SCH solution used for electrospinning was performed using a Malvern Zetasizer NS (Malvern Instruments, Malvern, UK). The particles size (average diameter), distribution (polydispersity index) and zeta potential were determined according to ISO 13321/1996 standard. The solution (1.5 wt %) was diluted three times prior to analysis. Scanning electron microscopy (SEM) analysis was carried out using a QUANTA 200 scanning electronic microscope with integrated EDX system, GENESIS XM 2i EDAX with Super Ultra-Thin Windows detector (FEI Company, Hillsboro, TX, USA). The fiber and particle diameter distributions for the electrospun samples were obtained from the SEM images by measuring the diameters of the fibers and particles for a surface of 5 μm × 5 μm. The range of measured diameters was divided into intervals of 10 nm size and the number of the diameters falling in each size interval was plotted against the size interval. To facilitate the visualization the plotted data was smoothed using adjacent-averaging. Atomic Force Microscopy (AFM), performed with a Solver-Pro-M type instrument (NT-MDT Company, Moscow, Russia) under ambient conditions, using standard tips of Si_3_N_4_ (10 nm curvature radius). The root mean square roughness was calculated on the total image sample after a second-order flatness treatment of the raw data. NT-MDT Nova v.1.26.0.1443 software was used for acquisition and analysis of the images. Transmission Electron Microscope (TEM) was carried out with a Hitachi High-Tech HT7700 microscope (Hitachi, Tokyo, Japan) (high contrast mode at 100 kV accelerating voltage) on electrospun grids (300 mesh holey carbon coated copper grids). X-ray photoelectron spectroscopy (XPS) spectra were recorded with a PHI 5000 VersaProbe (Physical Electronics, Chanhassen, MN, USA) spectrometer, equipped with a monochromatic Al X-ray source (1486.7 eV) of 100 µm beam size diameter under operation conditions typical for this technique (pressure in the sample chamber ~10^−7^ Torr). Measurements were taken at a take-off angle of 45° with respect to the sample surface. MultiPak V8.2C software was used for background subtraction, peak integration, and quantitative chemical analysis.

### 2.4. Contact Angle and Surface Free Energy Measurements

The static contact angles for the polymer meshes were determined by the sessile drop method, at room temperature and controlled humidity, within a maximum of 20 s after placing a 1 μL drop of liquid on the film surface, using a CAM-200 instrument (from KSV-Instruments, Helsinky, Finland). The average of at least 10 values at different locations on the surface was calculated. To obtain the total surface free energy and its components for the polymer meshes, the contact angles at equilibrium between the film surface and three pure liquids: twice distilled water, formamide, and di-iodomethane were measured by fitting the drop profile with the Young-Laplace equation [31]. The total surface free energy and the components were calculated using the acid base approach of van Oss and Good [31] which divides the total surface free energy into dispersive Lifshitz–van der Waals interaction (γ^LW^_s_) and polar Lewis acid–base interactions (γ^AB^_s_) (Equation (1)). The acid base interactions are subdivided into electron donor (γ^-^_s_) (Lewis base) and electron acceptor (γ^+^_s_) (Lewis acid) parts.

(1) $${(1+\cos\theta )\gamma }_{l}^{\mathrm{TOT}}=2\left(\sqrt{\gamma_{s}^{\mathrm{LW}}\gamma_{l}^{\mathrm{LW}}}+\sqrt{\gamma_{s}^{+}\gamma_{l}^{-}}+\sqrt{\gamma_{s}^{-}\gamma_{l}^{+}}\right),$$

where θ is the contact angle, γ^TOT^_l_ is the total surface tension of the liquid, and γ^LW^_l_ and γ^LW^_s_ are the polar Lifshitz–van der Waals components of the liquid and the solid, respectively, whereas γ^+^_s_, γ^−^_l_ and γ^−^_s_, γ^+^_l_ are the Lewis acid–base contributions of either the solid or the liquid phase as indicated by the subscripts. To solve the resulting system of equations it is necessary to use at least three test liquids with known γ^TOT^_l_, γ^LW^_l_, γ^−^_l_, and γ^+^_l_ values [34].

Blood compatibility is dictated by the manner in which the material surfaces interact with blood constituents, like red blood cells and platelets. When blood is exposed to a biomaterial surface, adhesion of cells occurs and the extent of adhesion decides the life of the implanted biomaterials; thus, cellular adhesion to biomaterial surfaces could activate coagulation and immunological cascades [35]. Therefore, cellular adhesion has a direct influence on the thrombogenicity and immunogenicity of a biomaterial, thus dictating its blood compatibility. The materials with lower work of adhesion would show a lower extent of cell adhesion than those with a higher work of adhesion. Polymer interactions with red blood cells are mediated mostly by the hydrophobic interactions with the lipid bilayer (the red blood cell hydrophobic layer containing transmembrane proteins) while the electrostatic interactions of the polymer with the surface charges are mediated by the direct interaction with membrane proteins, depending on the polymer characteristics [35].

The surface free energy of biomaterials and the corresponding values of the work of spreading can be used as characterization parameters for predicting cell spreading onto their surfaces and hence, for establishing their blood compatibility [35,36]. As work of adhesion measures the ease with which cells can adhere, determination of the work of adhesion for different cells can help a biomaterial scientist to predict the manner in which blood cells would react when they are exposed to a biomaterial. Generally, materials which exhibit lower work of adhesion would lead to a lesser extent of cell adhesion than materials with a higher work of adhesion [37].

Work of spreading of red blood cells (*W*_s/rbc_) and platelets (*W*_s/p_) were calculated according to Equation (2) [37]:

(2) $$W_{s}=W_{a}-W_{c}=2(\sqrt{\gamma_{\mathrm{sv}}^{\mathrm{LW}}{\times \gamma }_{\mathrm{lv}}^{\mathrm{LW}}}+\sqrt{\gamma_{\mathrm{sv}}^{+}{\times \gamma }_{\mathrm{lv}}^{-}}+\sqrt{\gamma_{\mathrm{sv}}^{-}{\times \gamma }_{\mathrm{lv}}^{+}})-{2\gamma }_{\mathrm{lv}},$$

where *W*_s_ is the work of spreading (i.e., the negative free energy associated with spreading liquid over a solid surface); *W*_a_ is work of adhesion (defined as the work required to separate the liquid and solid phases) and *W*_c_ is work of cohesion (defined as the work per unit area produced in dividing a homogeneous liquid).

### 2.5. In Vitro Drug Release Study

This was performed using a 708-DS Dissolution apparatus coupled with a Cary 60 UV-Vis spectrophotometer (Agilent Technologies, Santa Clara, CA, USA). The analyzed samples were electrospun onto the stainless steel basket used to hold the sample. The deposition time was 120 min. In order to reproduce the conditions of drug release when an antibacterial topical agent (SDZ) is applied in a wound dressing, a phosphate buffer solution of pH 6 was used as dissolution medium, at 50 rpm (37 °C). Aliquots of the medium of 10 mL withdrawn at predetermined time intervals were analyzed at λ_max_ of 260 nm, the characteristic absorption wavelength for SDZ [38]. The drug concentrations were calculated based on the calibration curve determined at the same wavelength. Sulfadiazine (SDZ) release kinetics was determined based on the semi-empirical equation proposed by Korsmeyer-Peppas [39].

## 3. Results and Discussion

### 3.1. Characterization of the Hybrid Nanostructures

**Figure 2:**
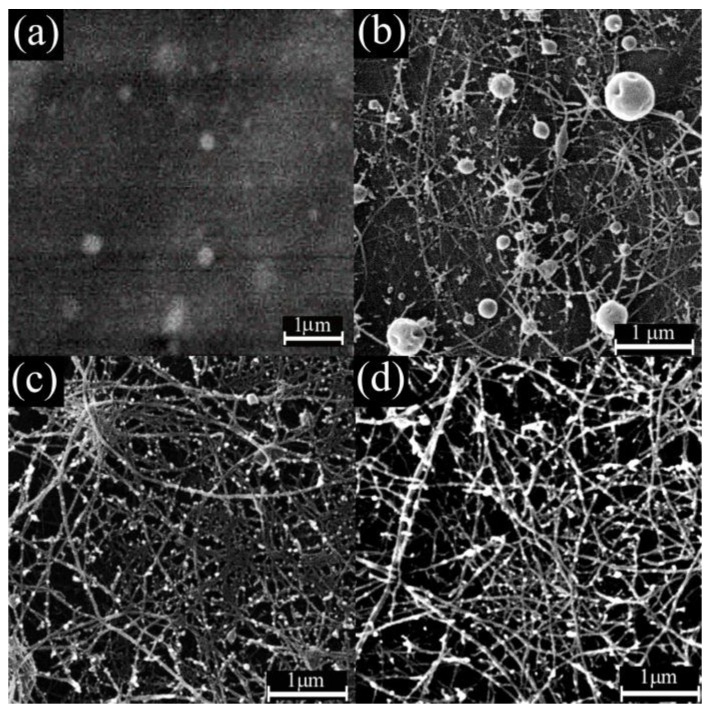
Scanning electron microscopy (SEM) images of (**a**) sulfadiazine modified chitosan electrospun alone SCH; (**b**) SCH and chitosan (CH) separately electrospun through the inner and outer nozzle respectively (SCH-IN); (**c**) SCH and CH separately electrospun through the outer and inner nozzle respectively (SCH-OUT); (**d**) chitosan/sulfadiazine mixture (CH/SDZ) electrospun alone.

**Figure 3:**
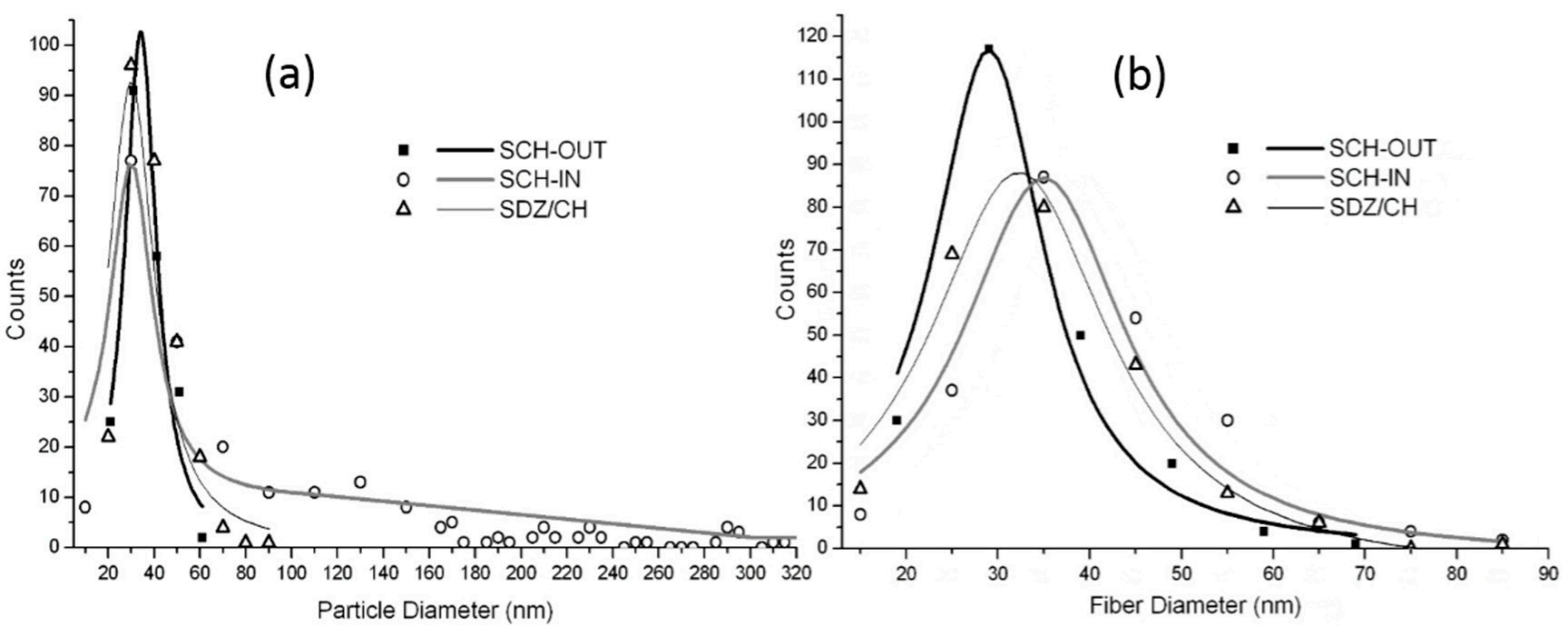
Particle (**a**) and fiber (**b**) size distributions for SCH-IN, SCH-OUT and SDZ/CH samples (to facilitate the visualization the data points were interpolated).

The SEM images reveal that the SCH solution electrospun alone (Figure 2a), in the same conditions as the other samples, produced only particles (electrospraying). The absence of fibers in the morphology is caused by the low SCH solubility in the acetic acid solution which determines the low degree of SCH chain entanglements and also the presence of SCH particles in the SCH suspension. In order to obtain a nanoporous fibrous mat, the SCH may be electrospun in combination with chitosan with high molecular weight. For the coaxial SCH-IN sample the SEM images (Figure 2b) reveal a hybrid particle/fiber morphology with a narrow distribution (Figure 3a) of particles (35 ± 10 nm) stuck onto the outside of the fibers (fiber diameter 32 ± 10 nm). In addition to this distribution the particle diameter histogram shows the presence of many larger particles with diameters up to 300 nm which can be explained by the low electrospinnability of the inner SCH solution. In this case, the SCH particles fed through the inner nozzle break the integrity of the outer CH forming fiber in the early stage of jet forming thus hindering fiber formation.

For the coaxial SCH-OUT and mono-axial SDZ/CH samples (Figure 2c,d), the SEM images reveal also a hybrid morphology with a high number of NP stuck/attached on nanofibers with diameters in the 30–40 nm range for both nanofibers and nanoparticles (Table 2). For these two samples the NF mesh is much denser in comparison with that resulting from the coaxial SCH-IN sample.

The absence of big particles and the presence of the much denser nanofiber mesh indicate that for the coaxial SCH-OUT sample, the integrity of the forming inner CH fiber is not disturbed by the SCH outer suspension during the fiber stretching, the SCH NP remaining attached onto the CH NF (as bright particles in the SEM image—Figure 2c). Similar hybrid morphology with nanofibers covered with metal-oxide nanoparticles [10] was obtained by other authors by coaxial electrospinning with the NF forming polymer solution flowing through the central nozzle and the NP suspension through the outer nozzle.

The same observations stand for the mono-axial SDZ/CH sample. In spite of the SDZ segregation to a high degree out of the CH fiber (bright particles in the SEM image—Figure 2d) the integrity of the CH fiber is maintained during the electrospinning of the SDZ/CH mixture. The segregation of SDZ at the NF surface is caused by the nanoscale dimensions of the NF and by the comparable diameters for the NF and SDZ particles. This leads to SDZ particles either incompletely encapsulated, or not centered in the fiber.

A similar process by which particles suspended/dispersed in the electrospun solution blend do not hinder fiber formation during emulsion/blending electrospinning and which produced a similar morphology was reported for inorganic BaTiO_3_ particles dispersed in polyvinyl alcohol and ethylcellulose. The BaTiO_3_ particles (100–300 nm) did not hinder fiber formation but were scattered along the polymeric fiber. Some of the particles were encapsulated into the polymeric nanofibers but many of them surrounded the nanofibers [11].

Thus, it can be concluded that the low solubility of SCH in acetic acid solution (for the coaxial samples) and SDZ in the SDZ/CH suspension (for the mono-axial sample) explains the presence of the NP on the surface of the CH nanofibers in the morphology of the studied samples (especially for SCH-OUT and SDZ/CH). For the coaxial samples, the presence of chitosan backbone in both the outer and inner electrospun solutions would help the sticking of the SCH nanoparticles onto the CH nanofibers [14]. The SCH and SDZ nanoparticles on the surface of the CH nanofibers are expected to be easily released when they are in contact with a wound, facilitating the wound healing process. In addition to the SDZ and SCH nanoparticles stuck to the CH nanofibers the transmission electron microscopy (TEM) images reveal the presence of SCH nanoparticles confined as beads into the CH nanofibers (discussed later in this paper).

The nanofiber diameter distributions (Figure 3b) are similar for SCH-IN, SCH-OUT and SDZ/CH samples because the main part of the nanofibers contains pure chitosan. So it can be concluded that for the studied samples the nanofiber morphology is controlled by the chitosan electrospinnability.

Therefore by combining the SCH and SDZ suspensions with the electrospinnable (nanofiber forming) CH, a hybrid CH nanofibrous structure can be produced which contains SDZ and SCH as active NP attached to the CH nanofibers. As it was mentioned in related studies reported in the same field, the NF structure helps tissue regeneration and provides pathways for the wound fluids to move out from the wound as well as allowing the transport of oxygen to the wound promoting the breathability [40]. Concomitantly, the NP entrapped (with a narrow size distribution—Figure 3a) into the NF mesh at the fiber surface [17] assures the burst release which can deal with the initial infection risk in the wound.

**Figure 4:**
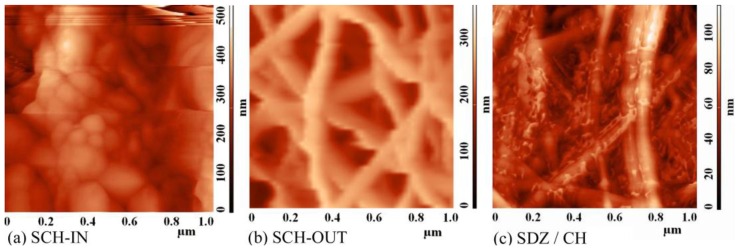
Atomic Force Microscopy (AFM) images of (**a**) SCH-IN; (**b**) SCH-OUT and (**c**) SDZ/CH samples (scanned area 1 μm × 1 μm).

**Figure 5:**
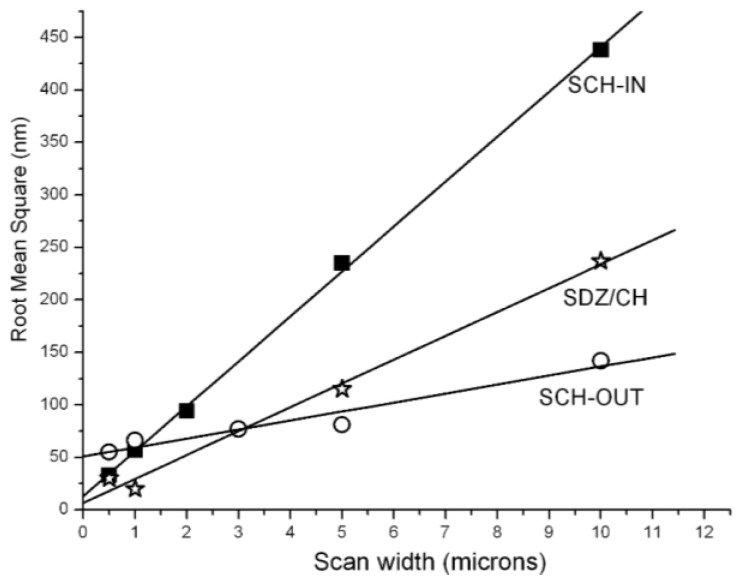
Root mean square roughness (RMS) dependence on the scan width for SCH-IN, SCH-OUT, and CH/SDZ samples.

The AFM images (Figure 4a,b) confirm the formation of the nanofiber mesh for SCH-OUT and SDZ/CH samples in accordance with SEM results, the aspect of nanofibers being rough because of stuck nanoparticles. For all the samples the roughness increases with the dimension of the scanned area (Figure 5). For scanned AFM areas higher than 2 μm × 2 μm the highest roughness is obtained for the SCH-IN sample while for smaller scanned AFM areas the roughness of the three samples becomes comparable (going under 50 nm). This is again in agreement with the SEM results which show the presence of large particles (with diameters up to 300 nm) in the 10 μm × 10 μm SEM images of the SCH-IN sample.

When AFM scanned small areas (500 nm × 500 nm) such big particles were avoided which resulted in much lower roughness for the SCH-IN sample, closer to the roughness of the SCH-OUT and SDZ/CH samples. Thus, at nanoscale the roughness of the SCH-IN, SCH-OUT, and SDZ/CH samples become similar.

**Figure 6:**
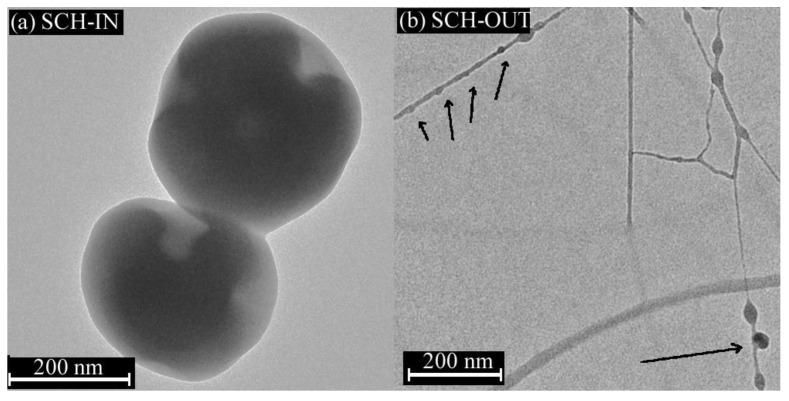
Transmission electron microscopy (TEM) images SCH-IN (**a**) and SCH-OUT (**b**).

For the SCH-IN sample the TEM images reveal that at least a part of the SCH particles fed into the inner nozzle are encapsulated/surrounded by a very thin layer (10–20 nm) of chitosan (Figure 6a). Due to the presence of the inner SCH particles the chitosan fibers are broken in the early stage of jet forming, leaving a great part of the SCH particles encapsulated with chitosan. For the SCH-OUT sample, the TEM images show thin nanofibers (10–15 nm) and SCH nanoparticles (15–35 nm) stuck, or even confined, as beads along the CH nanofibers. Similar to the SEM results, the TEM images show also SCH particles attached eccentric to the chitosan fiber (Figure 6b). During the electrospinning process of the SCH-OUT sample the integrity of the inner chitosan fiber is not disturbed by the SCH phase during fiber stretching. Thus, the SCH nanoparticles remain either confined in or attached to the CH nanofiber.

The elemental atomic composition determined from XPS data confirms the TEM results. All samples have as main surface elements carbon, oxygen, sulfur, and nitrogen. The elemental atomic composition differs from one sample to another regarding the content of each element. The detected sulfur atomic content was higher than the XPS typical detection limit (parts per thousand) [41] and was found to be higher for the SCH-OUT than for the SCH-IN sample (Table 3). Thus, it is expected that for the SCH-IN sample at least a considerable part of the SCH particles are encapsulated into a chitosan shell, this conclusion being consistent with the TEM results.

### 3.2. Contact Angle and Surface Free Energy Measurements

The mono-axial SDZ/CH sample is characterized by a moderate wettability (a water contact angle value of 76.5°) while the coaxial SCH-OUT sample is strongly hydrophobic (water contact angle 115°) due to the presence of water insoluble sulfadiazine units onto the outer SCH containing nanoparticles. The SCH-IN sample has a water contact angle (92°) at the boundary of the hydrophilic/hydrophobic region. This value is higher than the value corresponding to chitosan (~75°) [42] due to higher roughness for this sample [43]. All the samples are characterized by higher Lifshitz–van der Waals interactions than acid base interaction (Table 4).

As observed from Table 4 the total free surface energy decreases for coaxial electrospinning of sulfadiazine modified chitosan. This change of the surface free energy may lead to the decrease of the bio-adhesion ability of the material [44]. The polarity of a biomaterial surface significantly influences the type of interaction with tissues [45]. For this reason the polar component of the surface free energy was calculated. The contribution of the polar component to the total surface energy is higher for the SCH-OUT than for SCH-IN sample, meaning that sulfadiazine modified chitosan (SCH), which has at least three more polar groups (–NH_2_, –NH–, =SO_2_), is present on the surface of the nanofibers.

Compared to the electron acceptor component γ_sv_^+^, the electron donating part of the surface free energy γ_sv_^−^ is much higher for the S-CH-IN sample and lower for the S-CH-OUT sample. Thus, for the SCH-IN sample the surface is monopolar, behaving as a Lewis base while for the SCH-OUT sample the surface behaves predominantly as a Lewis acid because sulfadiazine is a sulfonamide with electron withdrawing groups as those containing nitrogen or substituted aromatic rings. Together with XPS results this is another confirmation of at least partial encapsulation of the SCH derivative with CH for the SCH-IN sample and also a confirmation of the presence of the SCH containing NP on the surface of fibers from the SCH-OUT sample.

Data of Table 4 reveal generally positive values for the work of spreading of red blood cells, *W*_s/rbc_, (except for SCH-IN sample) and negative values for the work of spreading of platelets, *W*_s/p_, suggesting a higher work of adhesion, comparatively with that of cohesion for the red blood cells, and a lower work of adhesion, comparatively with the work of cohesion for platelets.

Blood platelets are essential in maintaining hemostasis, being very sensitive to changes in the blood microenvironment. Platelet aggregation is used as a marker for the thrombogenic properties of the materials, the polymer-platelet interaction being an important step for understanding their hemocompatibility. The method for determination of surface free energy and the corresponding values of the work of spreading for biomaterials is used by many authors as “in silico” for predicting cell spreading onto their surfaces and blood compatibility [37,46]. Therefore, considering the exposure to blood platelets, the negative values of the spreading work indicate that all compositions of sulfadiazine-chitosan electrospun materials evidence cohesion, this result suggests that polymer samples do not interact with platelets, thus preventing activation of coagulation at the blood/biomaterial interface. All these characteristics are beneficial for wounds healing. In further studies cell cultures, bacterial cultures, platelet adhesion, and hemolysis tests will be performed.

### 3.3. In Vitro Drug Release

**Figure 7:**
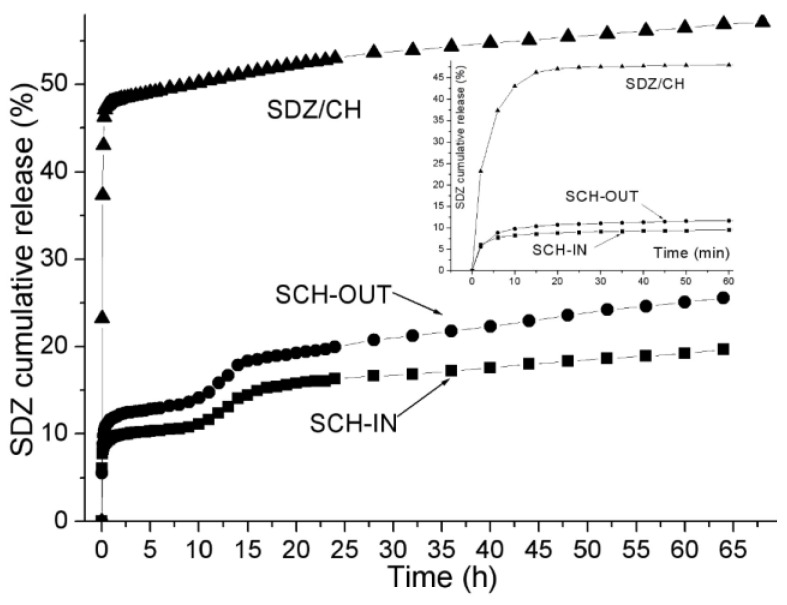
Cumulative sulfadiazine release (*M_t_*/*M*_∞_) profiles in phosphate buffer solution of pH 6 at 37 °C (where *M_t_* is the cumulative amount of drug released at time *t* and *M*_∞_ is the maximum amount released at the plateau of the release curve).

A local antibiotic release profile should exhibit a bi-phasic release behavior with a high initial release rate (burst release) [16], especially in the early inflammation stage in the wound healing process [47] followed by a sustained release phase at an effective inhibitory level. During the first hours of the wound treatment, it is essential to release a relatively high quantity of an antimicrobial drug in order to eliminate the immediately invading bacteria [47]. Later on, the continued low release rate should keep the wound “infection-free” for days to months [48]. The cumulative release profiles of SDZ from the studied electrospun meshes show a burst release occurring in the first 20 min, with ~47%, ~10% and ~13% SDZ released from the SDZ/CH, SCH-IN and SCH-OUT samples respectively. The burst is followed by a sustained gradual, slow release of the drug—measured up to 64 h (Figure 7).

An initial burst release was also observed in the case of electrospun drug loaded fibers prepared with ciprofloxacin hydrochloride encapsulated in chitosan/poly(vinyl alcohol) and chitosan/poly (caprolactone) nanofibers for which a 90% release of the drug in the first 25 min was reported [49]. As literature data shows, the burst release from the hybrid NF/NP structures can be associated with the segregation of the active compound on the fiber surface [17], due to the low drug/polymer compatibility causing drug accumulation on the fiber surface [50] or due to the large amount of free drug remaining non-encapsulated within the NF mesh [51].

In our case the burst release is caused by the detachment/desorption of the SCH and SDZ nanoparticles from the high specific surface area of the NF/NP morphology, as was also shown by Gan and Wang for proteins in CS nanoparticles [52]. There are also SCH and SDZ nanoparticles not enveloped with chitosan phase and thus not efficiently stuck to the CH nanofibers mesh as this was found also for tetracycline hydrochloride release from poly(ε-caprolactone) and biphasic calcium phosphate composite membranes [51].

Additionally, for the mono-axial SDZ/CH sample, the burst effect can also be explained by the high amount of SDZ in the electrospun suspension above the solubility limit. Other authors have reported an increased burst amount from 10% to 30% when the Ag-SDZ concentration increased from the solubility limit to twice this value, while the final plateau (after 160 h) was almost similar (60% and 70%) for the two samples [53].

After the initial burst, the SDZ release profiles have different shapes and released amounts for the coaxial samples (SCH-IN and SCH-OUT) compared to the mono-axial SDZ/CH sample.

While the SDZ/CH sample had a single stage release profile (burst + zero-order), the coaxial samples (SCH-IN and SCH-OUT) had a two stage drug release: After the initial burst there followed a short plateau (2–6 h) and then a second “burst release” (10–15 h) followed by a zero-order sustained release.

A second release stage was reported by other authors for several drug releasing systems. For chitosan based drug carriers, Dutta et al. [54] showed that the second release stage can be correlated with the CH swelling and that the time interval of the second release stage corresponds with the maximum swelling degree of the CH. In our case, the second release stage is caused probably by the release of the SCH nanoparticles strongly stuck onto the CH nanofibers due to presence of CH backbone in both the inner and outer coaxially electrospun solutions. This second stage release of the SCH NP takes place only when the CH is swollen enough to allow SCH nanofibers to detach from the CH nanofibers.

For this second stage, the release kinetics was evaluated—Table 5—using a modified [55] form of Korsmeyer–Peppas equation which takes into account both the lag time (delay) (*T*) and the burst effect where an initial mass (*M*_b_) has been already released *b = M*_b_*/M*_∞_ corresponding to the beginning of the second stage release:

*M*_(*t − T*)_/*M*_∞_ − *b* = *k*(*t − T*)*^n^* (3)

where: *M_t_* is the cumulative amount of drug released at time *t* and *M*_∞_ is the maximum amount released at the plateau of the release curve, *k* (in *h^−n^*) is the release constant, and *n* is the power law diffusion exponent describing the release mechanism.

For the SCH-OUT and SCH-IN samples, the second stage was considered to start after 6 h (*T* = 6 h), time at which the release was already ~13% and ~10% respectively. The diffusion exponents (*n*) thus found are 1.8 and 2.1, which shows that in this second stage the drug release is controlled by both diffusion and the relaxation of the polymer chains (super case II transport) (Table 5). In this transport mechanism, the diffusion of the entrapped drug out of the matrix starts after the chains are relaxed [56]. The release constant is twice higher for the release from SCH-OUT than from SCH-IN due to the difference in the morphology of the samples.

Regarding the released amount, the SDZ/CH sample released ~60% SDZ, while the coaxial samples (SCH-IN and SCH-OUT) had significantly lower (~20% and ~25%) release in the same time interval of 65 h (Figure 7). The higher total released amount from the mono-axial SDZ/CH sample as compared with the coaxial samples can be explained by the lower adhesion of the SDZ containing nanoparticles to the CH mesh. While in the mono-axial sample the SDZ molecules are physically dispersed into the CH mesh, in the coaxial samples, the SDZ is chemically linked to the CH backbone, forming the SCH phase. Thus, the presence of CH backbone in both the SCH and CH electrospun solutions increases the adherence of the SCH nanoparticles onto the final CH nanofibers in the latter case [15].

The release profiles show that the last stage, with constant release rate (zero-order release) begins after ~15 h, for both mono-axial SDZ/CH (~10 µg/day) and coaxial samples (~8 and ~5 µg/day for SCH-IN and SCH-OUT respectively). In this last stage, the SDZ drug from the nanoparticles well confined/entrapped into the CH nanofibers is released. The release rate is controlled by the erosion/disruption of the CH nanofibers as was shown in other studies. Ge et al. studied the release of various drugs from the chitosan matrix and the fate of chitosan in the release medium and found also a chitosan release from its microspheres immersed in PBS (pH = 7.4) which followed a slow (3% in 35 days) zero-order release mechanism [57]. Other authors demonstrated that the drug release depends on matrix degradation [52]. Based on the presented results it can be concluded that the remaining drug will be released along with the erosion/disruption of the chitosan.

The release rate in this last stage, i.e., in the range of 5–10 µg/day is enough to assure the minimum inhibitory concentration for various bacteria. The nanofiber meshes used in the release experiments had a surface of ~2 cm^2^ and contained 500 µg SDZ (for SDZ/CH sample) and 250 µg SDZ (for the coaxial samples). If it is considered as an exudate layer with a thickness of about 1 mm in contact with the wound dressing and if the release takes place in the moist layer between the dressing and the wound, a released volume of 0.2 mL corresponding to this surface of the studied meshes is obtained. In this case, the release rate of ~5–10 µg/day can be converted to ~25–50 µg/(mL·day) which is in the range of the minimum inhibitory concentration necessary for example for Gram-negative *Neisseria Meningitidis* (10 µg/mL) [58], *Neisseria Lactamica* (64 µg/mL) [59] and Gram-postive *Tropheryma Whipplei* (1 µg/mL) [60] bacteria established by other authors.

## 4. Conclusions

In this work, chitosan (CH) nanofibrous structures containing sulfadiazine (SDZ) or sulfadiazine modified chitosan (SCH) in the form of functional nanoparticles were obtained by mono-axial and respectively coaxial electrospinning. The mono-axial design consisted of the SDZ/CH mixture solution being fed through a single nozzle (SDZ/CH sample) while the coaxial design consisted of SCH and CH solutions separately supplied to the inner and outer nozzle (or in reverse order) (SCH-IN and SCH-OUT samples). The CH ability to form nanofibers assured the formation of a nanofiber mesh, while the SDZ and SCH, both in form of suspensions in the electrospun solution, assured the formation of the active nanoparticles which remain entrapped in the CH nanofiber mesh after the electrospinning process.

For the mono-axial sample (SDZ/CH sample) and the coaxial sample with the SCH suspension fed through the outer needle (SCH-OUT sample), the SEM images reveal a dense NF mesh with nanoparticles stuck onto the nanofibers. For these two samples the diameters for both nanofibers and nanoparticles were in the 30–40 nm range.

For all studied samples the cumulative release profiles of SDZ show a burst release occurring in the first 20 min followed eventually by a sustained gradual, slow release of the drug. The remaining SDZ would be released along with the errosion/disruption of the CH matrix or of SCH containing nanostructures.

## Figures and Tables

**Table 1:** Optima electrospinning parameters used.

Parameter	Value
Feed rate (for both the sulfadiazine modified chitosan (SCH) and chitosan (CH) solution)	0.7 µL/min
Needle-collector voltage	15 kV
Needle to collector distance	9 cm
Deposition time	120 min

**Table 2:** Dimensions of the morphological units identified in scanning electron microscopy (SEM) images.

Sample	Average Particle Sizes (nm)	Average Fiber Diameter (nm)
SCH and CH separately electrospun through the inner and outer nozzle respectively (SCH-IN)	40 ± 10 (narrow distribution). Also, there are large particles (up to 300 nm)	32 ± 10
SCH and CH separately electrospun through the outer and inner nozzle respectively (SCH-OUT)	35 ± 10	30 ± 10
chitosan/sulfadiazine mixture (CH/SDZ) electrospun alone.	40 ± 10	35 ± 10

**Table 3:** Atomic elemental composition of studied samples.

Sample	C (at %)	O (at %)	N (at %)	S (at %)
SCH-IN	64.1	30.7	4.9	0.2
SCH-OUT	68	26.6	5	0.4
SDZ/CH	60.9	30.8	7.8	0.5

**Table 4:** Surface tension parameters and work of spreading (mN/m) for red blood cells and platelets of uniaxial and coaxial electrospun sulfadiazine-chitosan samples.

Samples	γ_sv_^LW^	γ_sv_^ab^	γ_sv_^+^	γ_sv_^−^	γ_sv_^TOT^	*W*_s/rbc_	*W*_s/p_
SDZ/CH	43.5	3.4	3.3	0.9	47.0	30.3	−88.3
SCH-OUT	26.4	10.6	6.9	4.0	37.0	24.2	−105.6
SCH-IN	30.1	1.1	0.07	4.7	31.2	−4.1	−110.5

**Table 5:** Kinetic parameters of SDZ release from coaxially electrospun SCH-OUT and SCH-IN samples at pH 6. Modified Korsmeyer-Peppas model, second release stage.

Sample	*M*_∞_ (%) (after 65 h)	*b* (%)	*n*	*k* (h^−1^)	*R*^2^
SCH-IN	20	1/2	2.13 ± 0.05	0.04	0.99 ± 0.06
SCH-OUT	25	13/25	1.84 ± 0.08	0.1	0.99 ± 0.09
